# Effect of Laterally Moving Tactile Stimuli to Sole on Anticipatory Postural Adjustment of Gait Initiation in Healthy Males

**DOI:** 10.3390/brainsci13101411

**Published:** 2023-10-04

**Authors:** Hiroshi Kunimura, Hitoshi Oda, Taku Kawasaki, Ryo Tsujinaka, Naoki Hamada, Shiho Fukuda, Masakazu Matsuoka, Koichi Hiraoka

**Affiliations:** 1 Graduate School of Rehabilitation Science, Osaka Metropolitan University, 3-7-30 Habikino, Habikino 583-8555, Osaka, Japan; hiroshihayabusa821@yahoo.co.jp (H.K.); hito_pt23@yahoo.co.jp (H.O.); t.kawasaki@hakuho.ac.jp (T.K.); w12relax@gmail.com (N.H.); vqnw01142@ares.eonet.ne.jp (S.F.); m_matsuoka0815@yahoo.co.jp (M.M.); 2 Department of Physical Therapy, Todaiji Ryoiku Hospital for Children, 406-1 Zoshicho, Nara 630-8211, Nara, Japan; 3 Graduate School of Comprehensive Rehabilitation, Osaka Prefecture University, 3-7-30 Habikino, Habikino 583-8555, Osaka, Japan; eernvv4487@yahoo.co.jp; 4 School of Medicine, Osaka Metropolitan University, 3-7-30 Habikino, Habikino 583-8555, Osaka, Japan

**Keywords:** moving tactile stimuli, gait initiation, tactile sensation

## Abstract

This present study examined the effect of the laterally moving tactile stimuli (LMTS) to the sole on the anticipatory postural adjustment (APA) of the gait initiation. Thirteen healthy males participated in this study. A sound cue was provided at the beginning of each trial. The participants took three steps forward from a quiet stance at their preferred time after the start cue. The LMTS were delivered to the sole after the start cue. The loci of the tactile stimuli moved from the left- to the right-most side of the sole and then moved from the right- to the left-most side of that in a stimuli cycle. The duration of one stimuli cycle was 960 ms, and this cycle was repeated 16 times in a trial. The APA did not onset at the specific direction or phase of the LMTS, indicating that they did not use any specific phase of the stimuli as a trigger for initiating the gait. The LMTS decreased the amplitude and increased the duration of the APA. Simultaneously, the LMTS increased the time between the APA onset and toe-off of the initial support leg, indicating that they moved slowly when initiating gait during the LMTS. Those findings are explained by the view that the suppression of the APA induced via the LMTS to the sole is caused by the slowing down of the gait initiation due to masking the tactile sensation of the sole.

## 1. Introduction

There are several previous findings that prove moving tactile stimuli induced specific cortical activity. For example, the hMT+/V5 and inferior parietal cortex contributed to the perception of the moving tactile stimuli [1,2]. Transcranial magnetic stimulation over the primary sensory cortex or V5/hMT+ reduced the accuracy of discriminating the direction of the moving tactile stimuli to the fingertip [3].

Interestingly, a previous finding indicated that the moving tactile stimuli influence postural control in a quiet stance; the moving tactile stimuli to the sole, mimicking the tactile sensation of the sole during gait, caused lateral body sway in accordance with the phase of the moving tactile stimuli in quiet stance [4]. This means that the moving tactile stimuli to the sole interact with the postural control process. Anticipatory postural adjustment (APA) occurs prior to the motor output in postural tasks [5], and thus, APA is the event mediated via the postural control process. Thus, APA possibly interacts with the tactile perceptual process induced via the moving tactile stimuli to the sole.

Gait initiation is a postural task that involves APA. Thus, the moving tactile stimuli to the sole may change the APA of the gait initiation. The center of pressure (COP) moves to the initial swing leg side and backward immediately before the gait initiation, called the S1 period [6,7,8,9,10]. The COP displacement in the S1 period represents APA of the gait initiation [6,7,8,10]. The COP displacement in the S1 period was small in patients with Parkinson’s disease and has been considered one possible cause of the freezing of gait [11,12,13]. Thus, enhancing the APA with some intervention may be beneficial to improve the freezing of gait in patients with Parkinson’s disease. Repetitive cutaneous cues over the left hip abductor increased the COP displacement in the S1 period [14]. Rhythmic lateral weight shifting, rhythmic auditory cues or rhythmic arm swings increased the COP displacement in the S1 period [15]. Those previous findings indicate that rhythmic stimuli enhance the APA before the gait initiation. The laterally moving tactile stimuli (LMTS), in which the stimuli cyclically move from the left-most to the right-most side of the body and vice versa, are a kind of rhythmic stimuli. Therefore, the LMTS to the sole may enhance the APA before the gait initiation (Hypothesis 1).

Rhythmic lateral shifting of the body weight increased the APA amplitude before the gait initiation [15]. Rhythmic lateral shifting of the body causes the rhythmic change in the tactile sensation of the sole in the medial–lateral axis. Thus, the LMTS may mimic the tactile sensation of the rhythmic lateral weight shifting over the feet. If this view is true, the LMTS must enhance the APA as during the rhythmic lateral shifting of the body. If the APA is enhanced by the LMTS to the sole in healthy humans, it must be evidence suggesting that the LMTS is potentially useful for improving freezing of gait in patients with Parkinson’s disease.

The COP displacement in the S1 period for the gait initiation in response to a cue was greater than that for initiating gait at the participants’ own preferred time [7,13,14]. This means that the APA is greater when one initiates gait in response to an external cue. Accordingly, humans may use a particular phase of the LMTS as a trigger to initiate gait for enhancing the APA (Hypothesis 2). If this hypothesis is true, the APA must onset at a specific phase of the LMTS, and the APA amplitude must be greater when the LMTS are provided during gait initiation.

Monaural auditory cue to the ear contralateral to the preferred initial swing leg changed the probability of selecting the initial swing leg of gait initiation [16]. One possible explanation for this finding is that the selection process of the initial swing leg is influenced by the asymmetrical sensory input. The LMTS to the sole may also play a role for this asymmetrical sensory input. If this view is true, the LMTS to the sole influences the selection process of the initial swing leg of the gait initiation (Hypothesis 3). If this hypothesis is supported, then, the finding must be evidence for the LMTS to treat the across-trial variability of the initial swing leg selection in patients with Parkinson’s disease with freezing of gait [13]. We examined those three hypotheses.

## 2. Materials and Methods

### 2.1. Study Design

The type of the present investigation was an experimental study conducted on healthy humans in laboratory setting. The experiments were conducted at motor control laboratory of Osaka Metropolitan University in the term from August 2021 to August 2022. The participants initiated gait at their own preferred timing during the LMTS to the sole or without the LMTS. The order of the stimulation conditions was randomly assigned to each participant. The COP displacement in the S1 period, time events of the gait initiation, and the probability of the initial swing leg side were compared between the two stimulation conditions.

### 2.2. Participants

Thirteen healthy males aged 36.2 ± 2.5 years participated in this study. The number of participants was determined based on number of the participants in a previous study that found a significant effect of the moving tactile stimuli (*n* = 13) [2]. All participants had no history of neurological or musculoskeletal diseases. To confirm this, an experimenter asked whether the potential participants had a history of those diseases, and those who stated no history of the diseases were included in this study. There are gender differences in physical characteristics [17] and motor performance [18]. Thus, to exclude variability in motor performance caused by gender differences, only males were recruited. According to the Waterloo dominant foot test [19,20], two participants were left-footed, but the others were right-footed. The overview of this study was explained in a document, and written informed consent was obtained from all participants. The methods of this study were in accordance with the Declaration of Helsinki. The experimental design was approved by the ethics committee of Osaka Prefecture University.

### 2.3. Apparatus

**Figure 1:**
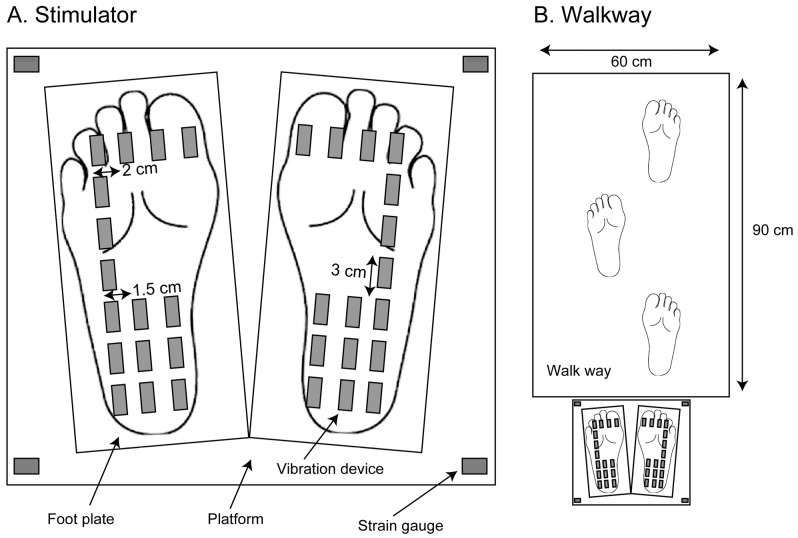
Vibration devices, foot plates, a platform under the feet (**A**), and a walkway in front of the stimulator (**B**).

A moving tactile stimulator (S-20008; Takei Kiki, Tokyo), which provided vibration stimuli to the sole, was placed over the ground (Figure 1A). A foot plate was placed under the participant’s foot while the participant maintained a quiet stance. Sixteen vibration devices were placed over each foot plate. The frequency of the vibration in each vibration device was 100 Hz. Strain gauges measuring the COP were placed on a rectangular platform under the foot plates. Each one of the strain gauges was placed at each corner of the rectangle platform, and the strain of the platform at each corner was measured. Using data from those four strain gauges, the COP was calculated. A walkway (width 60 cm, length 90 cm) was placed in front of this stimulator (Figure 1B).

### 2.4. Procedure

**Figure 2:**
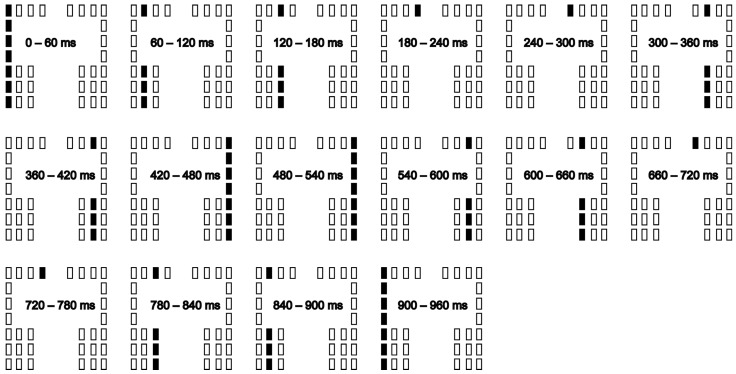
Time course of LMTS in a stimuli cycle.

The participants maintained quiet stance on the foot plates. The feet position was finely adjusted in each trial so that all the vibration devices were under the sole. Each trial lasted for 15 s. A sound cue was provided at the beginning of the trial, and the participants initiated gait at their preferred time within the time window of each trial and made three steps forward to the end of the walkway with their preferred velocity. The initial swing leg of the gait initiation was freely chosen by the participants in each trial. One of two conditions, gait initiation with (stimulation condition) and without stimuli to the sole (non-stimulation condition), was assigned in each trial. In the stimulation condition, the LMTS were given throughout the time window of the trial. The duration of each vibratory stimulus provided via the devices was 60 ms. The cycle duration of the LMTS was 960 ms, mimicking the cycle duration of the gait cycle, which is approximately 1 s [21]. The loci of the stimuli moved from the left- to right-most position of the sole and then moved from the right- to left-most position of that with 960 ms of one stimuli cycle, and this cycle was repeated 16 times (Figure 2). Thus, the stimuli lasted for 15 s. The stimuli were not given in the non-stimulation condition. Twenty trials were conducted for each condition. Taken together, 40 trials were conducted in each participant. One of the two conditions (stimulation and non-stimulation conditions) was randomly assigned in each trial. To achieve this, 40 random numbers were generated, and one of the two conditions was assigned to each random number, and then, the number was ordered from small to large. This ascending order of the random numbers generated was considered the order of the trial.

### 2.5. Variables

**Figure 3:**
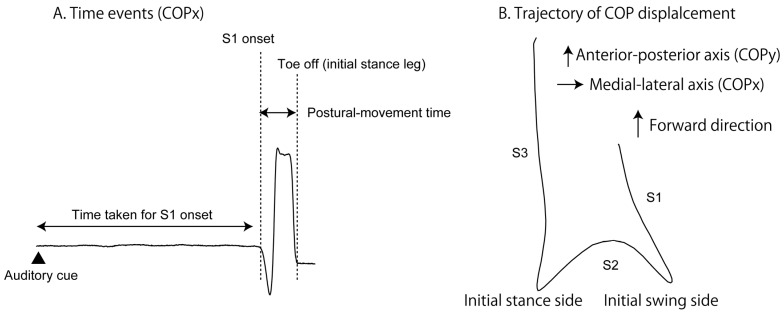
Data analysis. Time event on the COPx is shown in (**A**). The trajectory of the COP displacement during the gait initiation is shown in (**B**). Three portions of the trajectory are categorized in S1, S2, and S3 periods, respectively.

The COP displacement during the gait initiation is composed of three periods (S1, S2, and S3 periods; [9]; Figure 3B). The S1 period of the COP displacement was considered the APA [6,7,8,9,10]. The onset of the COP displacement was considered the onset of the S1 period. The first peak of the COPx displacement was considered the offset of the S1 and onset of the S2 period. The second peak of the COPy displacement was considered the offset of the S2 and onset of the S3 period. The offset of the COP displacement was considered the offset of the S3 period. The duration and amplitude of the COP displacement in each period were calculated. The probability of the initial swing leg side of the gait initiation was calculated.

Time events of the COP displacement are presented in Figure 3A. The duration between the start cue and S1 period onset (time taken for the S1 onset) was calculated. The time at the toe-off of the initial stance leg was defined as the moment at which the COPx displacement ended. The duration between the onset of the S1 period and toe-off of the initial stance leg (postural movement time) was calculated.

The mean and standard deviation (SD) of the COP position 0–100 ms before the S1 period onset was calculated. Those variables indicate preparatory status of postural control at the moment in quiet stance [15]. The SD of the COP represents the amount of the body sway [22,23], and mean COP represents the body position. The z-score of the mean COP was calculated as follows [4]. Mean COP averaged across the trials in both conditions (40 trials) was subtracted from mean COP in each trial. Then, the subtracted each COP was divided by SD of mean COP across the trials in both conditions (40 trials).

There were 16 phases of the LMTS (Figure 2). The LMTS from the left- to right-most position of the sole consisted of eight phases. The LMTS from right- to left-most position of the sole also consisted of eight phases. We categorized each trial into one of the 16 stimuli phases in which the S1 period onset corresponded to the time at the phase. Then, the number of trials categorized in each stimuli phase was counted.

### 2.6. Statistical Procedure

Paired *t*-test was conducted to examine the difference in means between the stimulation and non-stimulation conditions. Repeated measures of two-way ANOVA were conducted to test the main effect of the direction and phase of the stimuli on the number of trials categorized in each phase of the stimuli. The result of Greenhouse–Geisser’s correction was reported whenever Mauchly’s test of sphericity was significant. Bonferroni’s test was conducted as post hoc test. The alpha level was 0.05. All the statistical analyses were carried out using Excel Tokei ver. 3.20 (Social Survey Research Information, Tokyo, Japan). All the data in the Results were expressed as the mean and standard error of mean.

## 3. Results

### 3.1. COP before S1 Onset

**Figure 4:**
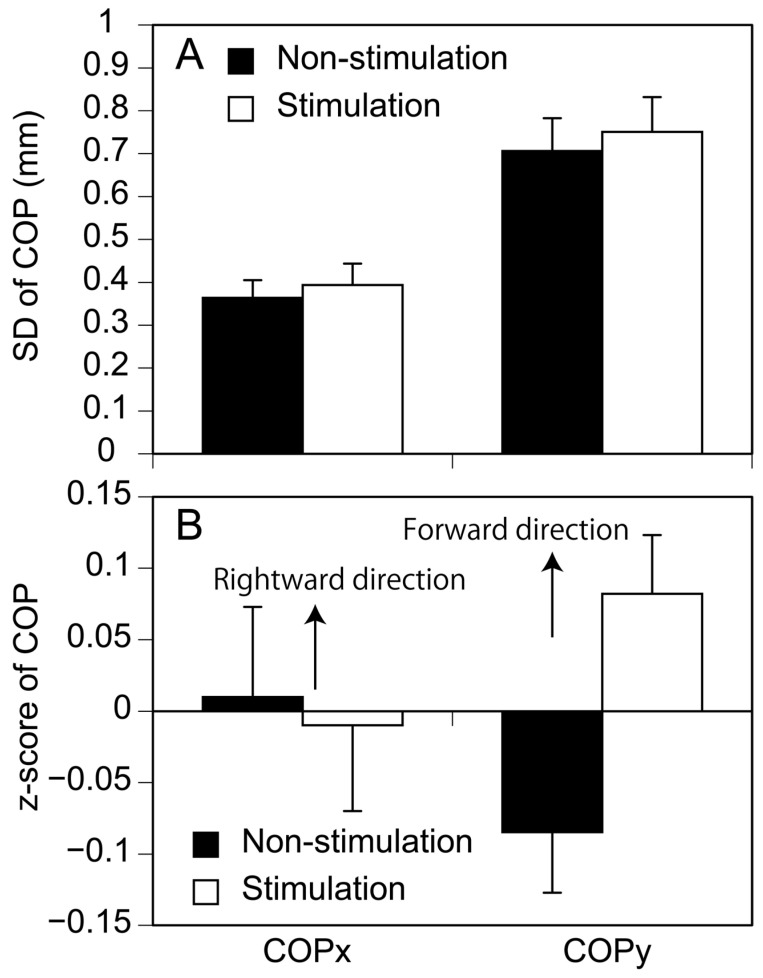
SD (**A**) and z-score (**B**) of COP position immediately before APA onset. Bars indicate mean and error bars indicate SD of mean.

SD of the COP, indicating the amount of the COP displacement in the time immediately before the S1 period onset is shown in Figure 4A. There was no significant difference in SD of the COP between the stimulation and non-stimulation conditions either in the anterior–posterior (*p* = 0.507) or medial–lateral axis (*p* = 0.210). The z-score of the mean COP, indicating the COP position, is shown in Figure 4B. There was no significant difference in mean COP between the conditions either in the anterior–posterior (*p* = 0.071) or medial–lateral axis (*p* = 0.876).

### 3.2. S1 Onset

**Figure 5:**
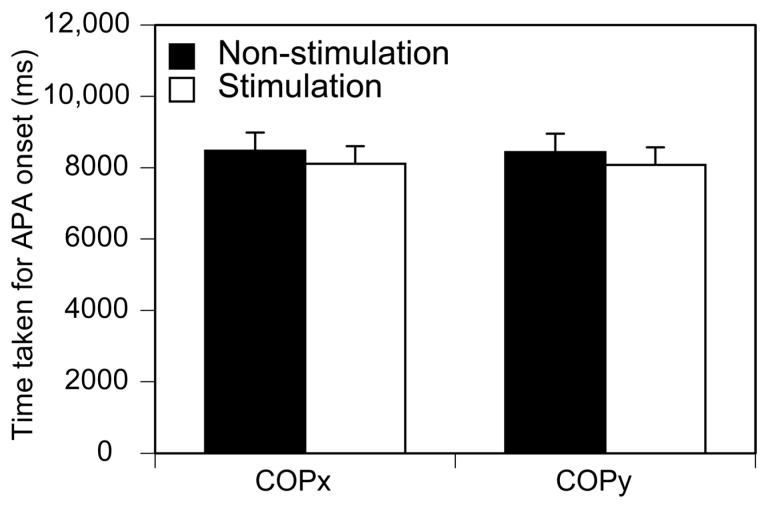
Time taken for APA onset. Bars indicate mean and error bars indicate SD of mean.

The time taken for the S1 period onset is shown in Figure 5. There was no significant difference in the time taken for the S1 period onset between the two conditions either in the anterior–posterior (*p* = 0.241) or medial–lateral axis (*p* = 0.246). There was no significant main effect for the direction [F (1, 12) = 0.823 *p* = 0.382] or phase [F (7, 84) = 1.135 *p* = 0.349] of the stimuli on the number of the trials in which the S1 period onset categorized in each stimuli phase. There was no significant interaction between those two main effects [F (7, 84) = 0.835 *p* = 0.561].

### 3.3. COP Displacement

**Figure 6:**
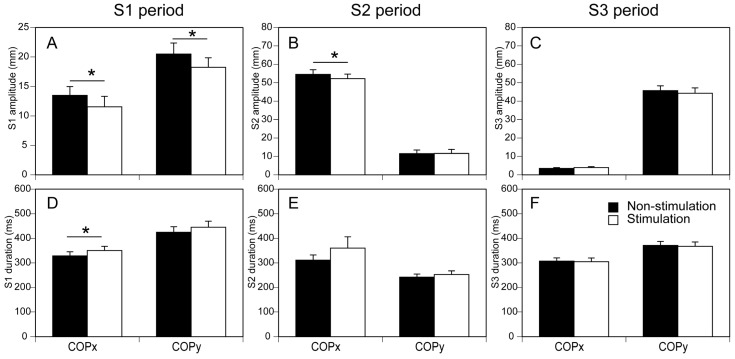
(**A**–**F**) Amplitude and duration of COP displacement in S1, S2, and S3 periods. Upper panels indicate amplitude, and lower panels indicate duration. Bars indicate mean, and error bars indicate SD of mean. Asterisks indicate significant difference (* *p* < 0.05).

The duration and amplitude of the COP displacement in the S1 period are shown in Figure 6A,D. The amplitude of the COP displacement in the stimulation condition was significantly smaller than that in the non-stimulation condition either in the anterior–posterior (COPy, *p* = 0.047) or medial–lateral axis (COPx, *p* = 0.030). The duration of the COP displacement in the stimulation condition was significantly longer than that in the non-stimulation condition in the medial–lateral axis (*p* = 0.014) but was not significantly different between the two conditions in the anterior–posterior axis (*p* = 0.224).

The duration and amplitude of the COP displacement in the S2 period are shown in Figure 6B,E. The amplitude of the COP displacement in the stimulation condition was significantly smaller than that in the non-stimulation condition in the medial–lateral axis (COPx, *p* = 0.004), but that was not significantly different between the two conditions in the anterior–posterior axis (COPy, *p* = 0.986). The duration of the COP displacement in the stimulation condition was not significantly different between the two conditions either in the anterior–posterior (COPy, *p* = 0.116) or medial–lateral axis (COPx, *p* = 0.148).

The duration and amplitude of the COP displacement in the S3 period are shown in Figure 6C,F. The amplitude of the COP displacement in the stimulation condition was not significantly different between the two conditions either in the anterior–posterior (COPy, *p* = 0.317) or medial–lateral axis (COPx, *p* = 0.309). The duration of the COP displacement in the stimulation condition was not significantly different between the two conditions either in the anterior–posterior (COPy, *p* = 0.416) or medial–lateral axis (COPx, *p* = 0.548).

### 3.4. Postural Movement Time

**Figure 7:**
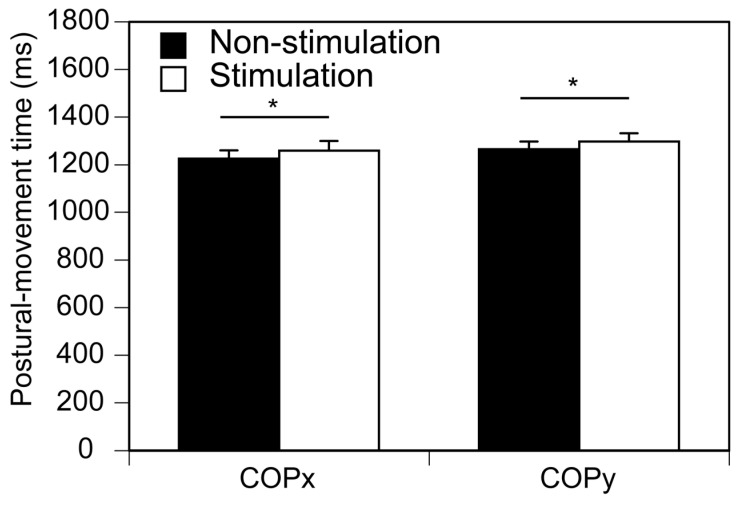
Postural movement time. The bars indicate mean and error bars indicate SD of mean. Asterisks indicate significant difference (* *p* < 0.05).

The postural movement time is shown in Figure 7. The time was 1226 ± 34 ms in the non-stimulation condition and 1258 ± 41 ms in the stimulation condition in the COPx. The time was 1265 ± 33 ms in the non-stimulation condition and 1296 ± 36 ms in the stimulation condition in the COPy. The time in the stimulation condition was significantly longer than that in the non-stimulation condition both in the medial–lateral (COPx, *p* = 0.033) and anterior–posterior axes (COPy, *p* = 0.048).

### 3.5. Selecting Initial Swing Leg

The participants initiated gait with the right leg in most of the trials both in the left- and right-footed participants. The participants initiated gait with the left leg with a probability of 0.007 ± 0.007 in the stimulation condition and 0.004 ± 0.004 in the non-stimulation condition. There was no significant difference in the probability of the gait initiation with the left leg between the conditions (*p* = 0.690).

## 4. Discussion

In this present study, we examined the effect of the LMTS on the sole on the APA, time events, and the probability of the initial swing leg side during the gait initiation. Hypothesis 1 (the LMTS to the sole enhances the APA before the gait initiation) was not supported, but rather, the APA was reduced by the stimuli. Neither Hypothesis 2 (humans use a particular phase of the LMTS as a trigger to initiate gait for enhancing the APA) nor Hypothesis 3 (the LMTS to the sole influences the selection process of the initial swing leg of the gait initiation) was supported.

### 4.1. COP Displacement in S1 and S2 Periods

The COP displacement in the S1 period was greater when the rhythmic lateral shifting of the body was performed before the gait initiation [15]. The rhythmic tactile sensation of the sole occurs during the lateral shifting of the body in stance. Thus, a potential explanation for this observed phenomenon could be that the rhythmic tactile sensation of the sole enhances the APA before the gait initiation. Accordingly, we hypothesized that the LMTS to the sole, which induces the rhythmic tactile sensation of the sole, enhances the APA before the gait initiation (Hypothesis 1). In spite of this hypothesis, the COP displacement in the S1 period was decreased via the LMTS to the sole. Thus, Hypothesis 1 was not supported.

The medial–lateral displacement of the COP in the S2 period was decreased via the LMTS. The S2 period is the moment at which the body weight shifts from the initial swing leg to the initial stance leg, as shown in Figure 3B [9]. The COP displacement in the S1 period, where the first COP displacement toward the backward and initial swing side occurs [9], is for shifting the body to the initial stance leg, which corresponds to the COP displacement in the S2 period [24,25]. Thus, the present finding is explained by the view that the decrease in the COP displacement in the S2 period is due to insufficient preparation of the weight shifting represented by the small COP displacement in the S1 period.

### 4.2. APA and Postural Movement Time

The postural movement time was extended when the LMTS were provided to the sole. The prolonged postural movement time reflects the slowing down of the gait initiation. The COP displacement in the S1 period increased as the velocity of the gait initiation increased [10,14]. The COP displacement in the S1 period interacted with the step movement of the gait initiation [26]. Accordingly, the decrease in the APA amplitude induced via the LMTS is likely caused by the slowing down of the gait initiation.

A likely explanation for the slowing down of the gait initiation induced via the LMTS to the sole is that the moving tactile stimulation to the sole masked the tactile sensation of the sole produced by the weight bearing over the feet. On the one hand, the tactile sensation of the sole occurs when humans maintain the stance because of weight bearing over the feet. On the other hand, the LMTS are artificial stimulation, and the spatio-temporal pattern of the stimuli is not related to the tactile sensation of the sole produced by the weight bearing over the feet in stance. Accordingly, it is possible that the LMTS functioned as noise for the tactile sensation of the sole produced by the weight bearing over the feet in stance.

It has been shown that masking the tactile sensation of the sole changes the body’s sway in a quiet stance. For example, the COP velocity and displacement in a quiet stance were increased when the tactile sensation of the sole was masked with the ischemic nerve block [27]. Acceleration of the lumber deviation in quiet stance was greater in patients with diabetic peripheral neuropathy who had impairment of tactile sensation [28]. Masking tactile sensation induced via the static tactile stimulus to the sole changed the body sway in a quiet stance [29]. The APA occurs at the quiet stance position before the gait initiation, and thus, the postural control in stance contributes to the APA before the gait initiation. Accordingly, the decrease in the size of the COP displacement in the S1 period and slowing down of the gait initiation induced via the LMTS to the sole may be explained by the view that the LMTS masked the tactile sensation of the sole, causing the change in the postural control process, leading to the slowing down of the gait initiation and negative impact on the APA.

However, this view must be handled with caution. If the slowing down of the gait initiation is caused by the masking of the tactile sensation of the sole, the body sway in stance immediately before the gait initiation must be influenced by the LMTS as occurring during masking tactile sensation of the sole in quiet stance in previous studies [27,28,29]. In the present study, as conflicting with the previous findings, the SD of the COP displacement before the S1 period was not significantly different between the conditions, as consistent with a previous finding that no significant change in the amount of the body sway was induced via the LMTS to the sole in quiet stance [4]. Accordingly, there is still an unresolved question to confirm the view that the decrease in the APA amplitude and the slowing down of the gait initiation are mediated by the mechanism underlying the effect of the masking tactile sensation of the sole in a quiet stance. Further studies are needed to determine whether the decrease in the APA amplitude and slowing down of the gait initiation induced via the LMTS is caused by masking tactile sensation of the sole.

### 4.3. Trigger of Gait Initiation

The COP displacement in the S1 period when initiating gait in response to a cue (e.g., auditory or tactile cue) was greater than that when initiating gait at the participants’ own preferred time [7,13,14]. This means that the APA is greater when humans initiate gait in response to an external cue (i.e., auditory or cutaneous cue). Accordingly, we hypothesized that humans may use a particular phase of the LMTS as a trigger to initiate gait (Hypothesis 2). If this hypothesis is true, the COP displacement in the S1 period must onset at a particular phase of the LMTS, and the APA must be greater when the LMTS are provided. The onset of the S1 period was not specific to a particular phase or direction of the LMTS, and the S1 amplitude was not significantly different between the two conditions. Thus, Hypothesis 2 was not supported. The LMTS were continuously provided throughout the trial. The loci of the stimuli moved to the adjacent column of the vibration devices each 60 ms (Figure 2), but there was no specific phase in which the stimuli in the phase were distinctively different from those in the other phases. Because of this, the participants must have perceived that the LMTS were not given serially in each phase but given as sequential stimuli that continuously moved. Thus, the negative finding may be explained by the view that the participants did not perceive the stimuli at each stimuli phase but perceived the moving stimuli as a sequence of the stimuli across the phases.

### 4.4. Selecting Initial Swing Leg

The probability of selecting the non-preferred leg as the initial swing leg of the gait initiation was as low as 0.004 to 0.007 in this present study. A previous study reported that the probability of selecting the non-preferred leg as the initial swing leg of the gait initiation ranged from 0.02 to 0.04 [16]. On the one hand, in this previous study, the participants initiated gait in response to a monaural auditory cue immediately. On the other hand, in this present study, the participants initiated gait at their own preferred time. Accordingly, the low probability of selecting the non-preferred leg as the initial swing leg in this present study is explained by the view that humans tend to select the preferred leg for the initial swing leg when the gait initiation is not hurried.

The variability of the initial swing leg side in the patients with Parkinson’s disease with the freezing of the gait was greater than that in the patients without the freezing of the gait [13]. Thus, an intervention that changes the selection of the initial swing leg side may be useful for the treatment of freezing of gait in those patients. Monaural auditory cue changed the probability of selecting the initial swing leg of gait initiation [16]. This indicates that the selection process of the initial swing leg side is influenced by the asymmetrical sensory input. Accordingly, the LMTS to the sole in the present study may play a role in this asymmetrical sensory input. Based on this view, we hypothesized that the LMTS to the sole influence of the selection of the initial swing leg side of the gait initiation (Hypothesis 3). The LMTS to the sole did not affect the selection of the initial swing leg. This means that laterally moving tactile sensation does not influence the selection process of the leg during gait initiation; Hypothesis 3 was not supported.

### 4.5. Limitation

In this present study, vibration devices were placed under the sole. There is inter-individual variability in the feet size, but the vibration devices were fixed to a foot plate. Because of this, the vibration devices were not always precisely in the same position under the sole among the participants, although the feet position was finely adjusted so that all vibration devices were under the sole. Accordingly, across-participant variability of the precise location of the vibration devices is the weakness of this present study. This present study was conducted on male participants. The motor performance in females is likely different from that in males [17,18]. Thus, the present finding obtained from male participants may not be applicable to females. Further studies are needed to confirm whether the present finding is applicable to females.

## 5. Conclusions

The LMTS to the sole decreased the APA amplitude before the gait initiation and made slower gait initiation. Those findings are explained by the view that the LMTS to the sole slows down the gait initiation due to masking tactile sensation of the sole, and this reduces the amplitude of the APA. This finding indicates that the LMTS to the sole plays a role in noise masking natural tactile sensation, and this noise may modulate the interaction between tactile sensation and postural response output.

## Data Availability

The data are unavailable due to ethical restrictions.
